# Enteritis Simulating Acute Intestinal Obstruction

**Published:** 1891-12

**Authors:** Daniel Parker

**Affiliations:** Calvert, Texas


For Daniel's Texas Medical Journal.

### ENTERITIS SIMULATING ACUTE INTESTINAL OBSTRUCTION.

BY DR. DANIEL PARKER, OF CALVERT.

[Read before the Austin District Medical Society December 17, 1891.]

THE FOLLOWING case, which occurred in my practice a number of years ago, will be made to serve as a foundation for what I shall have to say to you on this occasion, and although no notes were taken at the time, the instructive points were impressed upon my mind with sufficient distinctness and accuracy for the purposes contemplated in this paper.

In the month of July I was called to see a large, athletic negro, about 35 years of age. His health, until a few days previous to my visit, had been good, and the symptom for which he sought relief was inability to obtain any passage from the bowels. There was some tympanitis and tenderness over the abdomen, but the most striking symptom was weakness of the heart's action, cool skin, and an anxious expression of the countenance that indicated some grave lesion. His only complaint was of pain (tormina) in the bowels that he felt would be relieved by a fecal evacuation. This pain was intense, and the desire for a passage correspondingly urgent.

You all understand what July is to the agricultural negro. A paradise of hot sunshine and shade, the crop "laid by," and plenty of roasting ears and watermelons at hand. My patient had been reveling in these semi-tropical luxuries, and having had some experience in the impaction of watermelon seed and corn husks in the rectum, I too hastily diagnosed obstruction of the bowels from this cause, probably in the neighborhood of the ileo cæcal valve. My treatment was based on this diagnosis. I exhibited calomel, saline cathartics, large doses of sweet and castor oil by the mouth, and large enemas of warm water and castor oil, and applied warm baths and fomentations to the abdomen externally.

My patient soon vomited most of the substances taken by the mouth, and the enemas came away unchanged and unmixed. Prostration and collapse rapidly supervened, and death occurred on the third day of my attendance, with as marked symptoms of shock as could have been induced by a mortal lesion of some vital organ.

I had no doubt that death was caused by acute obstruction of some portions of the small intestines, either from internal strangulation in some form, intussusception or impaction of foreign bodies, and was decidedly inclined to attribute it to the latter cause. If I had seen the case in its incipiency, I might have arrived at a different conclusion, but at that time the diagnosis of *acute intestinal obstruction* seemed clear.

I regard it as extremely fortunate that I was able to obtain an autopsy, which, while it showed my diagnosis to be entirely at fault, and my treatment to be, at least, injudicious, gave me a very clear idea of a formidable pathological condition, which I believe I am right in saying is frequently not very well understood, and which has since served me, and my patients, a very good purpose.

It showed the *prima via* to be practically empty, from mouth to anus, and patulous throughout. The small intestines were somewhat distended with gas, and contained a little offensive fluid, about the consistency of gruel, in which a few watermelon seed, grains of corn and shreds of various substances were floating.

The trouble was apparent, however. The mucous and muscular coats of about two-thirds of the small intestines presented the lesions of intense inflammation. The blood vessels were highly injected, and the tissues of the bowel and corresponding mesentery were mottled in color between dark red and almost black. The line of demarkation between the sound and infected portions of the gut, was well marked, almost abrupt. The peritoneum, except that portion covering the inflamed segment of the intestines, was unaffected. There was no peritonitis, except such as was caused by contact. with inflamed tissues. It was a case of circumscribed enteritis, pure and simple, and it was now plain that the function of the muscular coat of a part of the intestines had peen destroyed by inflammation, and for that distance had been rendered a passive, flacid tube, destitute of propelling power. Of course no action of the bowels could be obtained. A glance at the crippled intestine explained everything.

I am inclined to think that the mistake I made is not an uncommon one, and that the frequency of obstructions of the bowels as a symptom of enteritis, is not sufficiently apprehended. This seems to be a type of the disease to the elucidation of which modern writers have contributed but little. So far as my researches go, with one exception, but slight reference is made to it in the recent literature of enteritis. If we extend our researches back, however, we are more fortunate.

The only paper I have been able to find devoted especially to its consideration, is a monograph by P. M. Kollock, M. D., of Savanna, Georgia, which appeared nearly fifty years ago,* entitled "Enteritis, with Cases, etc.," in which he describes very accurately the symptoms presented by my patient, and afterwards says: "The disease, in truth, is an enteritis or inflammation of

*American Journal Medical Sciences, April, 1844.

the small intestines, the muscular coat being involved occasions an arrest of peristaltic action, and the consequent obstinate constipation which is a prominent symptom of the case." It is a very interesting paper, and I shall have occasion to quote again from it further on.

Sir Thomas Watson, in his "Lectures on the Principles and Practice of Physic," which will always remain a classic among medical writings, and which are familiar to all of the older members of the profession, treats of all forms of intestinal obstruction* under the head of enteritis, and refers to the loss of contractile power of the intestine due to inflammation, as one cause of obstruction. He also makes some very valuable suggestions as to treatment, based on this pathological condition, and gives the careful student a better idea of its nature than most of subsequent writers, so far as I know.

Wood,† in treating of enteritis, mentions constipation as an occasional symptom, but devotes only a few lines to its consideration. Under the head of colic, or ileus, however, he gives a short and graphic account of the symptoms of the condition to which this paper refers, without mentioning enteritis as a cause.

Flint treats of enteritis without referring to constipation as a symptom, but under the head of "Functional Obstruction," or "Ideopathic Ileus,"‡ describes a condition not to be mistaken for any except the one under consideration.

Tanner simply mentions obstinate constipation as an occasional symptom of enteritis.

Johnson, in so voluminous and excellent a work as "Pepper's System of Medicine," makes no mention of such a condition as constipation in an extended article on "Acute intestinal catarrh," which term he considers more scientific than enteritis, while under the head of "Enteralgia"|| he recounts as symptoms in some cases collapse, with cool skin, distended abdomen, stercoraceous vomiting, etc., and while he does not refer to constipation or any inflammatory condition, there can be little doubt that these symptoms are more properly ascribed to the type of enteritis under consideration, or to some form of acute obstruction of the intestines.

---

*pp. 786–7, 3d American Ed.
†Vol. I, p. 617.
‡pp. 487–8, 5th Edition.
||Vol. II, p. 662.

John Ashurst, Jr.§ alone, of such recent authors as it has been my good fortune to examine, in the consideıation of acute intestinal obstruction, gives a connected and satisfactory account of enteritis as a cause of obstruction of the bowels.

It will not be profitable to consume further time in referring to authorities, which I think will strengthen the impression that while scant mention is made of obstruction of the bowels as a consequence of enteritis, under the various heads of ileus, iliac passion, ideopathic ileus, functional obstruction, colic and enteralgia, a disease has been described characterized by all the symptoms presented by my patient, viz.: intense, griping pain in lhe abdomen, notable depression, absolute inertia of the bowels, and occasional stercoraceous vomiting, without any reference to inflammation as a cause, while enteritis is generally treated with slight or no reference to any of these symptoms, except the first.

It seems clear to me that this is misleading, and that all of these terms, except colic or enteralgia, which should be restricted to non-inflammatory affections, should be expunged from the nomenclature of the profession, as they are intended to define a condition which evidently arises from inflammation of the mucous and muscular coats of the small intestines, and is, I think, correctly defined by the term enteritis.

I fear that this confusion of terms has borne fruit. For my own part, I recall at least one case, prior to the one narrated at the commencement of this paper, characterized by the same griping, twisting pain in the region of the umbilicus, the same looking forward to an action from the bowels as a sure source of re-relief, the same intense prostration, collapse and death, that I, in company with two other physicians, treated, and I speak for myself only when I say that to me it was simply a case of colic, ileus, or functional obstruction, according to the authors I consulted, and that I had no clear idea of the pathological condition with which I had to contend. Pephaps some of you can recall the case of a departed one who entrusted his life in your hands, and to whom the torture of so-called colic was the bodily antithesis of spiritual joys. If so, I am not alone in my experience.

Now, if a patient should come under my care, presenting the symptoms already so often enumerated, I would not say that he has colic, or ileus, or anything of that sort, but I would say that he has either enteritis that has destroyed the muscular power of

---

§Vol. VI, p. 50.

the intestine, or he has acute intestinal obstruction from internal strangulation, intussusception, or some kindred cause, bearing in mind that lead colic sometimes presents somewhat similar features.

The importance of a correct solution of this question cannot be exaggerated. In these days, when the abdomen is opened with such impunity, it is a matter of momentous consequence to be able to say whether strangulation, intussusception or any obstruction amenable to surgical treatment exists, or whether we have to deal with an inflammation in which the use of surgical means would be worse than useless.

The diagnosis once made, the way of the physician is comparatively clear. We would expect almost any case of enteritis to commence with griping pains in the abdomen, and diarrhœa, but these symptoms are so common that they are likely to be disregarded in the type under consideration, and the sufferer only applies for relief when agonizing tormina, great vital depression and inertia of the bowels have become so intense as to dominate all other symptoms.

The tormina is of the most distressing character, and is caused by violent and spasmodic contraction of the inflamed, but not yet disabled portion of the intestine, and its severity is readily understood when we take into account the abundant supply of terminal nerve filaments in the intestines, there being, according to Frey, "about two hundred ganglia belonging to the submucous, and over two thousand belonging to the mesenteric plexus, to be found to the square inch of intestine in the rabbit." The constant seat of this pain in the umbilical region is explained by the central location in the abdominal cavity of the mesenteric plexus, as well as the solar, from which it is mostly derived.

The vital depression, indicated by rapid, feeble pulse, sighing respiration, pinched and anxious expression of the countenance, and all the symptoms defined by the single word shock, constitutes the most marked feature of this condition, and I believe is more nearly diagnostic than any other. This is only what would be anticipated when we take into consideration the gravity of the lesion, the large extent of its surface and the intimate relation of the sympathetic nerve from which the intestines derive their principal nerve supply, with the systemic nerves, including the pneumogastric.

The inertia of the bowels, which is the symptom most likely to mislead, has been sufficiently considered, while occasional vomiting, elevation of temperature, tenderness, tympanites and dorsal decubitus with the knees drawn up, only need to be mentioned to be recognized as symptoms. These are all of a greater or less extent such as occur in acute obstruction of the intestines, and if any mistake in diagnosis is made, it will probably be on account of this similarity.

The more acute and urgent the symptoms of obstruction the more will they resemble those of enteritis, consequently cases of strangulation arising from entanglement in loops or bands of inflammatory origin or from internal hernia will present the greatest difficulty, and if a large portion of the intestine be included, and the strangulation be complete or nearly so, the symptoms may be very similar. We would hardly expect, however, that any case of strangulation would involve so large a surface as enteritis, or that the grave symptoms would supervene with equal rapidity. This remark refers, of course, to the stage and type of enteritis under consideration, viz: that where the inflammation is of sufficient gravity to destroy the muscular power of the intestine.

Volvulus should be accompanied by symptoms of less gravity, and although occurring suddenly and attended with complete obstruction, colicky pains, tenderness, etc., is not necessarily accompanied by rise of temperature, and the grave symptoms of prostration and collapse should occur later.

Intussusception occurs most frequently in early life, while this type of enteritis, according to my experience, is an affection of adult life. Intussusception, although presenting acute symptoms, is usually much more chronic in its course, bears much more resemblance to volvulus, and should present a tumor at the point of obstruction.

Obstruction from foreign bodies would hardly be mistaken for so acute an affection as enteritis, and need not be considered.

The history of the case will be likely to throw much light on the diagnosis. Enquiry will probably develop, in cases of enteritis with obstruction, the fact of exposure, the ingestion of in-indigestible food, or imprudent eating or drinking, preliminary diarrhœa, malaise, etc., while other forms of obstruction have no distinct prodrome. It is true that a history of previous inflammation would tend to confirm the diagnosis of strangulation, or a history of constipation, that of volvulus or obstruction from foreign bodies, but it would probably be too remote and indefinite to have much weight.

All the cases of this character that I have seen occurred in hot weather. I think we can apprehend a reason why this season should be favorable for its development. The relaxation due to protracted high temperature enfleebles the digestive organs, while the lower temperature of the night, and that caused by too rapid evaporation of perspiration in a draught cause an arrest of cutaneous transpiration as well as a feeble cutaneous circulation. This elimination and circulation both being vicariously assumed by the mucous membranes produces the pathological process, popularly termed "taking cold;" and if the blow should fall on the mucous membrane of the bowels while loaded with indigestible matter, some type of enteritis is a probable result.

If the diagnosis is once satisfactorily made, the treatment should present no special difficulty. As before intimated, however, here is where the trouble lies. The two authors before referred to, who give the most satisfactory account of this condition, concur in the opinion that mistakes in diagnosis fraught with serious consequences are of frequent occurrence. Kollock in the monograph before quoted from says: "The disease is peculiar by reason of the extremely various views which are generally entertained in regard to it, the consequent erroneous and unsuccessful treatment based on these views, and the happy effect of treatment based on entirely different views."

Ashurst,* under the head of treatment of intestinal obstruction, says: "There is probably no class of cases so habitually mismanaged by otherwise intelligent practitioners as that under consideration;" and gives as a reason for this the comparative rarity of their occurrence, and the consequent unfamiliarity of physicians with their symptoms and pathology. If I am to judge by my own experience I shall have to accept the impeachment of these writers as valid, and the subject of this paper was chosen with the idea in my mind that, perhaps, there were others who had thought as little on this subject as I had for several years of my professional life.

In the treatment of this type of enteritis, opium in some form easily leads all other remedies in importance. It controls peristalic action, thus giving the bowels much needed rest, and allays the pain which is a powerful factor in producing the condition of

*Vol. VI, p. 60, International Cyclopedia of Surg.

depression and collapse. Both from therapeutic and euthanasic considerations it is a great boon to the patient. It should be exhibited until rest is obtained, and large doses will probably be required.

The practice of blood letting in this disease has had some ardent advocates, notably Kollock, whose article was written especially to "exemplify the decidedly beneficial effects of blood letting." He recommends the abstraction of blood "without regard to quantity, but with a view to syncope;" and cites cases in which this treatment was pursued to the practical exclusion of all others, with the happiest results. Near the close of his paper he says: "In conclusion, I may be allowed to remark that I know of no disease of the same violence and danger to which a plan of treatment appears to be so perfectly adapted, and to be attended with such certain and invariable success as that we have been considering. So much in this the case that I have never yet had an opportunity of testing my pathological views in regard to this disease by an autopsical examination."

Watson, to illustrate the beneficial effects of venesection, narrates an incident in his personal history. He says, "I well remember, though it is many years ago, being myself badly treated for enteritis. Being ill in a strange place, I sent for the nearest practitioner, who happened to be a very ignorant man. Finding that I was sick, and that my bowels did not act, he gave me, for two or three days in succession, strong drastic purges, with no other effect than that of increasing my sickness, and adding to the abdominal pains I suffered. I was then seen by a most intelligent physician (this was before I had paid any attention to physic myself), and the first thing he did was to have me copiously bled, and the immediate effect of the bleeding was to send me to the night chair." These authors wrote, however, when blood letting was almost a universal panacea, and some allowance should probably be made on this account.

Ashurst, who represents the modern idea in regard to blood letting, says: "I have not employed general bleeding in these cases, but I should not hesitate to do so if leeches could not be procured. I have, however, seen the whole aspect of the case changed by the application ot leeches over the inflamed segment of the bowel." It seems to me the remedy is a rational one, and one that should be employed in some cases.

Warm fomentations and baths are useful, and should be employed ; upon this point all agree, while counter-irritation by means of mustard or other rubefacient is recommended by some.

Enemas, while they cannot relieve the inertia, may remove irritating substances, and thus help to limit the inflammation; and if vomiting should be a distressing symptom, lavage of the stomach, as recommended by some in acute forms of intestinal obstruction, may be attended with good results.

Alcoholic stimulants and heart tonics may be considered in connection with the symptoms of prostration.

Purgatives need only be mentioned to be condemned. They cannot fail to add to the distress of the patient and the gravity of the disease.

So much for the treatment when the diagnosis is clearly made out. It is quite possible, however, that some cases may occur where the element of uncertainty cannot be eliminated. On this head I cannot refrain from quoting again from Ashurst. He says: "I would strongly urge that the surgeon should not in any case of intestinal obstruction open the abdomen, unless he has been able to form some distinct notion of what he expects to find, or, at least, until he has been able to satisfy himself that there is positive mechanical occlusion;" and in the same connection he adds, "From my own experience I am led to believe that a large proportion of cases of intestinal obstruction met with in practice are really examples of enteritis, and that if recognized as such and promptly treated, the number of recoveries would be much larger than the present."*

While some in these days when laparotomy has become the "fad" might open the abdomen to remove a doubt, as well as an obstruction, if perchance one be found, for my part I should adopt the course recommended by Ashurst. In case of doubt I would treat my patient for enteritis, believing that I was giving him the best chance for his life.

